# Beeswax in Pharmaceutical Sciences: A Comprehensive Review of Its Chemical Composition, Functional Applications, Types, and Formulation Roles

**DOI:** 10.3390/ijms27083486

**Published:** 2026-04-13

**Authors:** Kampanart Huanbutta, Bajaree Chuttong, Khanchai Danmek, Pornsak Sriamornsak, Kittipat Suwanpitak, Tanikan Sangnim

**Affiliations:** 1 Department of Manufacturing Pharmacy, College of Pharmacy, Rangsit University, Pathum Thani 12000, Thailand; kampanart.h@rsu.ac.th; 2 Meliponini and Apini Research Laboratory, Department of Entomology and Plant Pathology, Faculty of Agriculture, Chiang Mai University, Chiang Mai 50200, Thailand; bajaree.c@cmu.ac.th; 3 Biotechnology Program, School of Agriculture and Natural Resources, University of Phayao, Phayao 56000, Thailand; khanchai.da@up.ac.th; 4 Faculty of Pharmacy, Silpakorn University, Nakhon Pathom 73000, Thailand; sriamornsak_p@su.ac.th; 5 Academy of Science, The Royal Society of Thailand, Bangkok 10300, Thailand; 6 Faculty of Pharmaceutical Sciences, Burapha University, Chonburi 20131, Thailand; kittipat.su@go.buu.ac.th

**Keywords:** beeswax, biomaterials, lipid-based excipients, drug delivery systems, controlled drug release, nanostructured lipid carriers, physicochemical properties, natural products

## Abstract

Background/Objectives: Beeswax, a complex natural secretion primarily derived from *Apis mellifera* and *Apis cerana*, has evolved from an ancient remedy into a multifunctional excipient and bioactive material in modern pharmaceutical sciences. This review evaluates its physicochemical properties, pharmaceutical applications, and emerging biomedical potential, while addressing current quality and regulatory challenges. Methods: A narrative review was conducted by analyzing literature on the chemical composition, functional properties, conventional uses, advanced drug delivery applications, pharmacological activities, and quality control of beeswax, emphasizing structural characteristics, formulation roles, and integration into innovative delivery technologies. Results: Beeswax is a lipid-based matrix composed of over 300 constituents, including wax esters, hydrocarbons, and free fatty acids, conferring thermoplasticity, biocompatibility, and structural stability. Traditionally, it functions as a stiffening agent, viscosity modifier, and emulsion stabilizer in topical formulations, forming an occlusive barrier that enhances skin hydration. In advanced systems, it serves as a solid lipid matrix in nanostructured lipid carriers (NLCs), microspheres, and 3D-printed tablets, enabling controlled drug release and improved bioavailability of lipophilic compounds. It also exhibits antimicrobial, anti-inflammatory, and wound-healing activities, while beeswax-derived policosanols show potential cardiovascular and gastroprotective benefits. However, concerns regarding paraffin adulteration and pesticide contamination highlight the need for stringent analytical and regulatory oversight. Conclusions: With rigorous quality control and sustainable sourcing, beeswax remains a versatile, eco-friendly material bridging traditional medicine and advanced pharmaceutical innovation.

## 1. Introduction

Natural products have perennially served as a vital source of therapeutic agents and excipients in medicine, with their utility spanning from traditional remedies to advanced pharmaceutical formulations. Among these, materials derived from the honey bee (*Apis mellifera*), particularly beeswax, have a rich history of use that dates back to ancient civilizations. Historically, beeswax was a key ingredient in ointments and balms for wound healing and skin protection, a practice documented in ancient Egyptian, Greek, and Roman texts, as well as in traditional systems of medicine like Ayurveda [1]. In contemporary pharmaceutical sciences, beeswax continues to be a valuable excipient, prized for its biocompatibility, low toxicity, and unique physicochemical properties. This long history of safe use is underscored by its regulatory acceptance; for instance, it is designated as Generally Recognized as Safe (GRAS) by the U.S. Food and Drug Administration (FDA), which facilitates its use in a wide range of products [2]. It is officially recognized in various pharmacopeias and is used in a wide array of dosage forms. Its complex lipid composition (see Section 3.1) imparts desirable physicochemical properties such as the ability to act as a stiffening agent, an emulsifier in water-in-oil (W/O) emulsions, a viscosity modifier, and a matrix-forming agent for controlled-release preparations [3]. These properties have solidified its role in the formulation of semi-solid preparations like creams and ointments, where it not only provides structure but also forms a protective, occlusive barrier on the skin [4].

Beyond these traditional roles, the application of beeswax has expanded into novel drug delivery technologies. Researchers are exploring its potential in formulating modern systems such as solid lipid nanoparticles (SLNs) and microspheres, which can enhance the bioavailability of poorly soluble drugs, offer targeted delivery, and provide sustained release profiles [5,6]. Furthermore, the inherent biofunctionality of beeswax, including its well-documented antimicrobial and anti-inflammatory properties, adds a significant therapeutic dimension. While historically perceived merely as a chemically neutral and inert excipient, the complex chemical composition of beeswax—comprising a delicate balance of monoesters, free fatty acids, and hydrocarbons—is, in fact, fundamental to its pharmaceutical applications. This intricate chemical makeup dictates its physicochemical behavior, transforming beeswax from a passive structural vehicle into a highly versatile functional excipient. Consequently, its specific chemical components actively interact within formulations to modulate drug release kinetics, enhance physical stability, and contribute to the structural integrity of advanced drug delivery systems [4,7]. Despite its widespread use, several challenges remain, including compositional variability, adulteration, and inconsistencies in quality control, which may affect reproducibility and performance in pharmaceutical formulations. In addition, existing reviews often focus on isolated aspects of beeswax, such as cosmetic applications or general properties, without fully integrating its roles across modern drug delivery systems (DDSs). Furthermore, the rapid expansion of advanced drug delivery technologies (e.g., nanostructured lipid carriers, microsystems, and 3D-printed dosage forms) necessitates a more structured and function-oriented evaluation of beeswax as a pharmaceutical excipient.

To address this gap, this review provides an integrated overview of beeswax, encompassing its chemical composition, classification, physicochemical properties, and multifaceted roles in pharmaceutical formulations. It bridges historical applications with contemporary uses in conventional dosage forms and emerging advanced DDS, offering a cohesive and up-to-date perspective for formulation scientists. Unlike previous reviews that predominantly emphasize cosmetic or apicultural aspects, the present review specifically integrates excipient functionality, advanced drug delivery technologies, biofunctional properties, and regulatory considerations within a unified framework. Ultimately, this review aims to provide a comprehensive analysis of beeswax, linking its intricate composition to its functional performance, while also identifying current limitations and future research directions.

## 2. Origin, Composition, and Types of Beeswax

**Figure 1:**
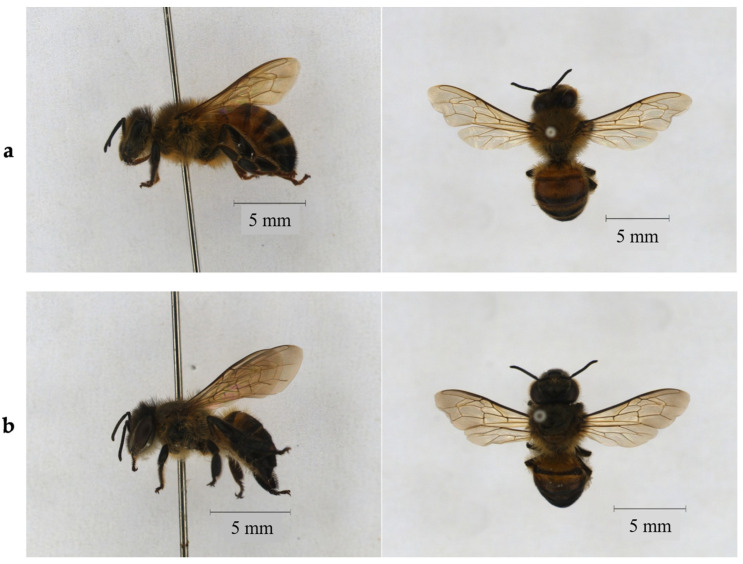
(**a**) *Apis mellifera* (Western honey bee) and (**b**) *Apis cerana* (Asian hive bee).

Beeswax is a valuable, complex natural secretion produced by young worker honey bees of the genus *Apis*, particularly *A. mellifera* (Western honey bee) and *A. cerana* (Eastern honey bee), secreted in liquid form from special wax glands in the abdomen, which hardens into scales used to build the honeycomb structure [7]. This structure serves multiple critical functions: for food storage (honey, pollen), for brood rearing, for thermoregulation, and as a mediator in chemical and mechanical communication within the colony [8]. *A. mellifera* has a larger body size comparing to *A. cerana*, with worker bees measuring about 12–15 mm, predominantly golden-brown with orange or brown banding, and queens up to 20 mm [9]. *A. mellifera* is the wax source most extensively studied, particularly concerning the physicochemical properties and detection of adulteration, which remains a growing issue globally. While *A. cerana* is generally smaller, slightly less robust, and shows less pronounced banding compared to *A. mellifera* (Figure 1). *A. cerana* is also one of the most commonly bred honey bee species from which commercially sourced beeswax is derived, its product, known as Ghedda wax, exhibits distinct chemical and physical properties compared to *A. mellifera* wax. Notably, Ghedda wax is characteristically less acidic, a difference supported by the finding that *A. mellifera* beeswax contains remarkably high levels of free fatty acids (up to 18%) compared to *A. cerana* (3.6%) [10]. Additionally, the wax of *A. cerana* has a melting point of 65 °C, which is approximately 2 °C higher than that of *A. mellifera* [11]. From a construction standpoint, *A. cerana* requires a smaller cell size for its comb building than the European races of *A. mellifera***.** However, A. mellifera can produce beeswax more than that of *A. cerana.* Both *A. mellifera* and *A. cerana* are recognized as promising sources for the extraction and purification of valuable long-chain primary aliphatic alcohols known as policosanols [12].

Beeswax intended for commercial use, such as recycled blocks and comb foundations, contrasts sharply with genuine or “virgin” beeswax (like wax cappings) in terms of purity and quality. Commercial beeswax is often subject to adulteration and contamination, which may affect quality and safety (see Section 6). This issue is exacerbated because commercial beeswax, often recycled from old combs and classified as an animal by-product, frequently lacks mandatory authenticity control prior to market placement. The recycling process itself involves melting old combs and cappings, which can concentrate pesticide residues (like lipophilic acaricides) and introduce non-wax impurities like cocoons and pollen, potentially resulting in compromised wax quality. In contrast, genuine beeswax (newly built comb or cappings) contains no impurities or residual contaminants, providing a vital reference standard against which commercial products are tested using established physico-chemical parameters defined by bodies like the International Honey Commission (IHC) and the European Pharmacopoeia (PhEur) [13,14,15].

## 3. Physicochemical Properties

The utility of beeswax in pharmaceutical sciences is fundamentally linked to its distinct physicochemical characteristics and the resulting functional properties it imparts to formulations [16,17,18]. This section delves into the chemical makeup of beeswax and connects it to its roles as both a versatile excipient and a bioactive material.

### 3.1. Chemical Composition

Beeswax is a complex natural product secreted by the glands of young worker bees, *A. mellifera*, to serve as a construction material for honeycombs. Chemically, it is a lipid-based mixture of over 300 different substances. Its exact composition can vary based on bee genetics, wax age, botanical origin, geographical origin, and processing conditions but its primary constituents are a rich blend of hydrophobic compounds. The major classes of chemical constituents in *A. mellifera* beeswax are presented in Table 1 [19]. Such variability of constituents in beeswax can influence key physicochemical parameters (e.g., melting range, acid value, ester value, and hydrocarbon content), which in turn affect formulation properties including viscosity, crystallinity, drug loading, and release behavior. Consequently, batch-to-batch differences in beeswax composition may impact reproducibility, particularly in lipid-based and advanced DDS. To mitigate these effects, pharmacopeial standards (e.g., PhEur, United States Pharmacopeia (USP)) define acceptable ranges for critical parameters, while analytical techniques such as Gas Chromatography–Mass Spectrometry (GC–MS) and Fourier-Transform Infrared Spectroscopy (FTIR) enable compositional profiling and quality control. Additionally, formulation strategies such as the use of purified or standardized grades and blending with other lipids can improve robustness and reduce sensitivity to compositional fluctuations.

Chemical parameters such as the acid value (AN), ester value (EN), and saponification number (SN) are critical for quality control of both white and yellow beeswax. As shown in Table 2, the PhEur 11.0 specifies ranges for AN (17–24), EN (70–80), and SN (87–104), with slight variations between white and yellow wax [13]. In comparison, the USP47-NF42 requires an AN of 17–24 and an EN of 72–79 for both wax types, but notably does not list a saponification value (SN), instead requiring a Saponification cloud test [20]. The Japanese Pharmacopoeia (JP XVIII) lists an AN of 5–9 or 17–22 and an SN of 80–100, but does not specify an ester value [21]. These parameters are used to determine the free fatty acid content and the proportion of esterified components, thereby ensuring the identity, purity, and overall quality of beeswax employed in pharmaceutical and cosmetic applications.

A significant concern in the beeswax market is economic adulteration, where cheaper, wax-like substances such as paraffin, ceresin, beef tallow, stearic acid, and carnauba wax are added to increase profit. The addition of these foreign substances degrades the physicochemical characteristics of the beeswax. For instance, adulteration with paraffin causes a decrease in the acid, ester, and saponification numbers, as paraffin lacks free or esterified fatty acids [14], while adding beef tallow or stearic acid can increase the saponification and acid numbers, respectively [22]. Although classical chemical tests can indicate adulteration, they may fail to detect low levels of contaminants (e.g., less than 5% paraffin). For more reliable detection, analytical techniques such as GC–MS and FTIR are used for compositional profiling and adulteration detection (see Section 6) [23].

### 3.2. Physical Characteristics

The enduring utility of beeswax in pharmaceutical sciences is fundamentally rooted in its unique and advantageous physical characteristics. The physicochemical behavior of beeswax (Section 3.1) underlies its functional performance that formulators have leveraged for centuries to create stable, effective, and cosmetically elegant dosage forms [19]. The physical profile of beeswax can vary slightly depending on its origin and processing, most notably between yellow beeswax (Cera Flava) and its bleached counterpart, white beeswax (Cera Alba).

#### 3.2.1. Thermal Properties

The thermal behavior of beeswax plays a crucial role in its function as a structuring and stiffening agent in semi-solid formulations. Unlike pure crystalline substances that exhibit a sharp melting point, beeswax melts over a range of temperatures, typically between 61 °C and 66 °C, due to its heterogeneous composition. It becomes plastic at around 32 °C, with a relative density of 0.96 at 15–25 °C and a refractive index of 1.44 at 75 °C [24]. This broad melting range is particularly advantageous for pharmaceutical manufacturing processes. It is high enough to ensure that the final product remains solid under ambient and physiological conditions, yet sufficiently low to allow easy melting and incorporation into formulations using standard heating equipment without excessive energy consumption. Upon cooling, beeswax solidifies at its congealing point, imparting the desired structure, texture, and consistency to creams and ointments. The processes of melting and solidification are therefore fundamental to achieving the optimal physical characteristics of the final product. Controlled heating and cooling cycles during manufacturing are essential to obtain a stable, homogeneous formulation and to prevent the development of a granular or brittle texture.

Beeswax demonstrates excellent thermal stability within the typical manufacturing temperature range of 70–80 °C (158–176 °F). However, prolonged exposure to elevated temperatures above 85 °C (185 °F) can result in thermal degradation, leading to discoloration (darkening), changes in odor, and alterations in its chemical and physical characteristics. When the wax is heated above 150 °C (302 °F), esterification occurs with a consequent lowering of acid value and elevation of melting point. At approximately 204 °C (400 °F), beeswax reaches its flash point, at which ignition and combustion may occur [25,26]. Therefore, precise temperature control during formulation and processing is essential to maintain the integrity, functionality, and quality of beeswax-containing products.

#### 3.2.2. Rheological and Structural Properties

The rheological properties of beeswax are perhaps the most important reason for its widespread use in topical preparations. It acts as a multifunctional excipient that profoundly influences the flow behavior, texture, and stability of formulations [27]. Beeswax is an excellent viscosity enhancer and stiffening agent. When added to oil-based or emulsion systems (e.g., creams and lotions), it increases the viscosity to the desired level. This provides the “body” or “substance” required for a topical product, ensuring it is thick enough for localized application without being difficult to spread. The concentration of beeswax can be precisely adjusted to achieve a wide range of consistencies, from soft, spreadable creams to firm, solid ointments and cerates [3].

Beeswax can work as structuring agent in emulsions. In W/O and oil-in-water (O/W) emulsions, beeswax helps to stabilize the system. In the external oil phase of W/O emulsions, it forms a crystalline network that entraps the dispersed water droplets, preventing coalescence and improving stability [28]. It also imparts a desirable non-greasy, smooth feel upon application. For occlusive and emollient properties, when applied to the skin, beeswax forms a fine, protective film. This film is occlusive, meaning it reduces transepidermal water loss (TEWL) from the skin, thereby promoting skin hydration. This makes it a valuable emollient for protecting and soothing dry, chapped, or irritated skin [4].

#### 3.2.3. Solubility and Compatibility

The solubility profile of beeswax dictates its use in various formulation types. Beeswax is lipophilic in nature. Beeswax is insoluble in water and aqueous solutions but is sparingly soluble in cold alcohol [20]. It is, however, soluble in most fixed and volatile oils, as well as in solvents like chloroform, ether, and warm carbon tetrachloride [29]. This lipophilic character makes it a natural choice for anhydrous (water-free) ointments and the oil phase of emulsions. Beeswax is highly compatible with a wide range of common pharmaceutical and cosmetic ingredients, including vegetable oils, mineral oils, fatty acids, and other waxes [30]. This versatility allows it to be easily integrated into diverse formulations without causing instability or incompatibility issues [19].

## 4. Pharmaceutical Applications of Beeswax

### 4.1. Conventional Application of Beeswax as Pharmaceutical Excipient

Beeswax is a natural substance with a long history of use in a wide array of products, including pharmaceuticals, owing to its desirable physicochemical properties [4]. As a pharmaceutical excipient, both yellow beeswax (cera flava) and white beeswax (cera alba) have established conventional applications, primarily in topical and oral dosage forms. Its functions range from acting as a stiffening agent and emulsion stabilizer to forming matrices for controlled drug release. The emulsion-stabilizing behavior of beeswax is governed by a combination of interfacial and bulk structural mechanisms at the molecular and supramolecular levels. Upon cooling, beeswax forms crystalline domains, predominantly in the β′ polymorphic form (orthorhombic packing), which can adsorb irreversibly at the oil–water interface and act as Pickering-type stabilizers. These solid lipid crystals create a rigid physical barrier around dispersed droplets, effectively preventing coalescence [31].In parallel, beeswax components, particularly long-chain esters, diesters, and fatty acids, undergo self-assembly within the oil phase to form a three-dimensional crystalline network (oleogel). This network increases viscosity and transforms the continuous phase into a semi-solid structure, thereby immobilizing dispersed droplets and reducing their mobility and collision frequency. The combination of interfacial crystal adsorption and bulk phase network formation results in enhanced mechanical stability and resistance to droplet aggregation [32]. This dual mechanism distinguishes beeswax from conventional surfactants and underlies its effectiveness as a hybrid stabilizer in W/O systems.

One of the most traditional uses of beeswax is as a stiffening and viscosity-increasing agent in topical formulations such as creams and ointments. It is incorporated at concentrations of 5–20% to impart the desired consistency and body to these semisolid preparations. Beyond simply modifying texture, beeswax serves a crucial role in W/O emulsions. Its presence allows for the incorporation of significant amounts of water into oily or paraffin bases, resulting in stable emollient creams that can enhance skin penetration and drug absorption [3]. Moreover, yellow beeswax is capable of forming a soap-like compound when reacted with borax [33]. Although beeswax is widely used in topical formulations, direct head-to-head studies comparing bioavailability with and without beeswax are limited. Available evidence is largely indirect, indicating that beeswax enhances skin hydration, reduces transepidermal water loss (TEWL), and improves barrier function, which are associated with increased skin retention rather than enhanced systemic absorption. Comparative clinical data suggest superior skin performance of beeswax-containing formulations relative to conventional creams, while formulation studies demonstrate sustained release behavior in beeswax-based systems. Overall, beeswax appears to modulate drug delivery by promoting prolonged residence time and controlled release rather than increasing immediate bioavailability [4,18,34].

In solid oral dosage forms, beeswax is utilized as a polishing agent for sugar-coated tablets, providing a characteristic luster to the finished product [3,35]. White wax is also employed to modify the melting point of suppository bases, ensuring their physical stability, particularly in warmer climates. Moreover, white wax is also used as a film coating in sustained-release tablets. Wax coating can also be applied to modulate drug release of drug from ion-exchange resin beads [3]. The impact of beeswax composition on drug release kinetics is primarily governed by its ester and hydrocarbon fractions, which influence matrix crystallinity and hydrophobicity. Higher ester content (≈35–70%) is associated with increased structural order and matrix cohesiveness, leading to slower diffusion and more sustained release profiles. In contrast, higher hydrocarbon content (≈12–18%) enhances hydrophobicity and reduces matrix polarity, further limiting drug permeability, particularly for hydrophilic compounds [36]. Disruptions in crystalline structure, arising from compositional variability or lipid blending, may increase molecular mobility and accelerate release. Although direct quantitative correlations between specific compositional ratios and release kinetics are not yet well established, studies indicate that relatively small variations in beeswax content (e.g., 10–15% *w*/*w* in lipid matrices) can significantly alter release behavior, including transitions from diffusion-controlled to near zero-order kinetics [18].

### 4.2. Application of Beeswax in Advanced DDS

Apart from its traditional use in ointments, emulsions, and other topical preparations, beeswax has recently attracted increasing interest in advanced DDS. Its biocompatibility [18], hydrophobic character [37], film-forming capability [38], and slow biodegradability make it a valuable excipient for the development of controlled-release formulations [39]. Modern drug delivery platforms have utilized beeswax to modulate drug release profiles [40], enhance physicochemical stability [31], improve bioavailability [41], and facilitate taste masking [42]. Furthermore, beeswax has been incorporated into micro- and nano-structured delivery systems [43], pharmaceutical films [44], and even emerging technologies such as 3D-printed dosage forms [40].

#### 4.2.1. Sustained Release of Solid Dosage Forms

Beeswax possesses hydrophobic characteristics that make it highly compatible with other excipients, allowing it to effectively modify or control DDS by regulating drug release rates, enhancing stability, and improving the overall performance of the formulation [45]. The study by Jaber, Al-Remawi, and Abdel-Rahem (2024) presents the development of a novel chitosan–beeswax matrix designed as a controlled-release gastro-retentive floating system for delivering curcumin as an adjuvant therapy against *Helicobacter pylori* infection. Using a hot melting process and response surface methodology, the researchers optimized formulation parameters—identifying ideal conditions as a mixing time of 3 min, melting temperature of 92.2 °C, and beeswax concentration of 13.3% (*w*/*w*). The optimized tablets exhibited excellent floating capacity in acidic media and followed a zero-order drug release pattern, ensuring sustained curcumin release [46].

#### 4.2.2. Microsphere and Microcapsules

**Figure 2:**
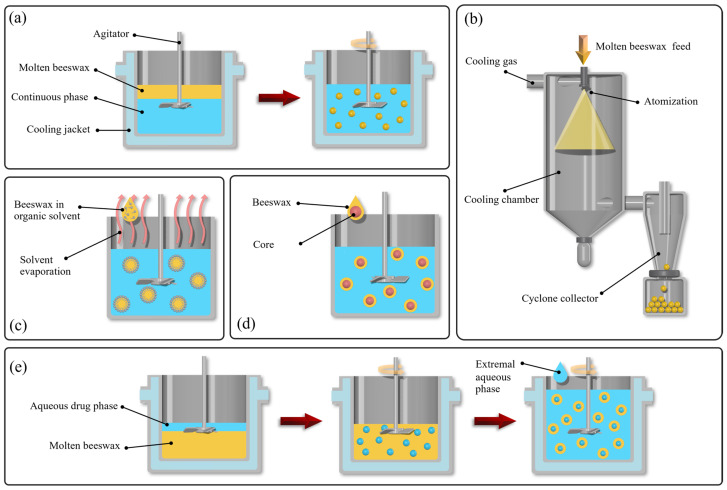
Schematic illustration of beeswax microsphere and microcapsule preparation methods: (**a**) melt dispersion, (**b**) emulsion congealing, (**c**) solvent evaporation, (**d**) coacervation–phase separation and (**e**) Double emulsion.

Beeswax has emerged as a versatile excipient in the design of microsphere and microcapsule-based DDS due to its hydrophobicity, non-reactivity, and ability to form stable lipid matrices [45]. These properties make it ideal for encapsulating both hydrophilic and lipophilic drugs [47,48], enabling controlled or sustained release, protection from degradation, and targeted delivery to specific tissues or organs [6]. Generally, there are several methods to prepare microspheres and microcapsules by beeswax, including melt dispersion, emulsion congealing, solvent evaporation, coacervation-phase separation, and double emulsion techniques as shown in Table 3 and Figure 2.

Overall, the choice of preparation method significantly influences particle size, encapsulation efficiency, and release behavior. Melt-based techniques are simpler and suitable for lipophilic compounds, whereas emulsion-based and solvent evaporation methods offer better control over particle size and drug distribution. These differences highlight the importance of method selection in tailoring beeswax-based delivery systems for specific therapeutic applications [18].

The 2024 study by Brahmi et al. developed single- and bi-layered beeswax-based microparticles (ranged from 70.2 µm to 133.7 µm) for colon-targeted mesalamine delivery, optimizing formulation parameters to achieve high entrapment efficiency (78.87%) and controlled release. Using ethylcellulose or cellulose acetate butyrate as outer coatings, the bi-layered systems provided pH-dependent delayed release (released at pH 7.4 for 90.64% at 24 h), ensuring mesalamine release primarily in the colon. The release followed a non-Fickian diffusion mechanism [54]. This anomalous transport indicates that the drug release is not governed solely by concentration gradients (Fickian diffusion) but also involves the structural relaxation or erosion of the beeswax-based matrix [55]. This further confirms beeswax’s role as a natural, effective matrix for site-specific drug delivery.

The benefit of beeswax microcapsules is not only for controlled release, but also it can be used to protect the substance inside from the environment. For example, Promita Gundev and her team used natural beeswax as an encapsulating material for butylated hydroxytoluene (BHT) due to the susceptibility of cold-pressed oils to oxidation. The beeswax encapsulation helps enhance oxidative stability, prevents rancidity, and increases the shelf life of the oil by providing higher thermal stability to the antioxidant [56].

#### 4.2.3. Nanostructured Lipid Carriers and Composite Systems

In the development of nanostructured lipid carriers (NLCs), beeswax serves as the solid lipid component, forming the structural core of the nanoparticle. Unlike Solid Lipid Nanoparticles (SLNs), which rely solely on solid lipids, NLCs incorporate a blend of solid (beeswax) and liquid lipids (oils). Beeswax-based NLCs enable co-delivery of multiple therapeutic agents (e.g., polyphenols [57]) and cosmetic agent (e.g., vitamin E [58]), with improved cellular delivery and reduced burst release as presented in Table 4. The main purposes of using beeswax in NLCs are to form a structural solid core, as beeswax serves as the solid lipid component that provides a rigid matrix to maintain the nanoparticle’s shape and physical stability [43]; to control drug release [58], as the solid beeswax matrix helps immobilize the drug and prevent sudden dose dumping while enabling sustained release over time; to enhance drug loading capacity [58], since blending solid beeswax with liquid lipids creates imperfections in the crystal lattice that allow more space to accommodate drug molecules such as polyphenols and vitamin E; and to improve cellular uptake, as the lipid nature of beeswax facilitates interaction with cell membranes and enhances the intracellular delivery of encapsulated agents.

Collectively, these studies demonstrate that beeswax-based NLCs provide enhanced drug stability, controlled release, and improved skin or cellular delivery. A consistent trend is the reduction in burst release and improved bioavailability of lipophilic compounds. The combination of beeswax with liquid lipids creates structural imperfections that increase drug loading capacity, highlighting its advantage over conventional solid lipid systems.

Beyond simple lipid nanoparticles, beeswax is increasingly utilized in composite systems where it is blended with polymers, inorganic materials, or hydrogels to create hybrid delivery platforms. For example, composite nanofibers were prepared from PVA and beeswax blends. The addition of beeswax increased fiber diameter and surface roughness while significantly enhancing thermal stability and slowing dissolution in physiological saline compared to pure PVA. These improved structural and thermal properties position the composite mats as promising biodegradable materials for biomedical applications such as wound dressings [62]. In the work of Narisara Ngamekaue and Pakamon Chitprasert, beeswax was incorporated with carboxymethyl cellulose to prepare an emulsion-based composite coating for holy basil essential oil-loaded gelatin microcapsules, which served to enhance shelf-life stability and facilitate targeted delivery to the distal small intestine [63].

#### 4.2.4. Other Application of Beeswax in Advanced DDS

Beeswax continues to demonstrate exceptional versatility in modern drug delivery technologies beyond its traditional functions as a stiffening or emulsifying agent [4]. Owing to its biocompatibility [64], biodegradability [65], and thermoplastic behavior [66], it has been integrated into a range of innovative systems designed for site-specific delivery, enhanced bioavailability, and controlled release. Table 5 summarizes representative examples of beeswax utilization across various advanced delivery platforms.

These examples (Table 5) illustrate the versatility of beeswax across diverse drug delivery platforms, ranging from oral and topical systems to advanced technologies such as microneedles and 3D printing. A key trend is its role as a multifunctional excipient, simultaneously contributing to structural integrity, controlled release, and biocompatibility, which supports its continued expansion in pharmaceutical innovation. Overall, the reviewed studies indicate that beeswax functions not only as a structural excipient but also as a key modulator of drug release, stability, and delivery efficiency. Its performance is strongly influenced by formulation type, concentration, and interaction with other lipids or polymers. However, variability in composition and the lack of standardized evaluation across studies limit direct comparison, highlighting the need for more systematic and quantitative investigations.

### 4.3. Biofunctionality

Beyond its role as an inert carrier, beeswax possesses inherent biological activities that can contribute to the overall efficacy of a formulation.

#### 4.3.1. Antimicrobial and Antifungal Activity

Beeswax exhibits intrinsic antimicrobial properties against various pathogenic bacteria and fungi, making it valuable for treating skin infections and preventing microbial overgrowth in formulations. Studies have demonstrated its effectiveness against Gram-positive bacteria (e.g., *Staphylococcus aureus*, *Streptococcus epidermidis*, *Bacillus subtilis*) and Gram-negative bacteria (e.g., *Escherichia coli*, *Pseudomonas aeruginosa*), as well as the yeast Candida albicans and the fungus *Aspergillus niger* [12]. These inhibitory effects are often enhanced synergistically when beeswax is combined with other natural products, such as honey and olive oil, proving effective in managing conditions like pityriasis versicolor and tinea infections [7]. Furthermore, beeswax-containing mixtures have demonstrated the ability to eliminate *C. albicans* in infants with diaper dermatitis [69].

#### 4.3.2. Wound Healing and Tissue Regeneration

Beeswax actively modulates tissue regeneration rather than simply covering the wound. In animal models treating second-degree burns, beeswax formulations have been shown to increase collagen formation, neovascularization, and fibroblast activity [70]. A proposed mechanism for this bioactivity involves the upregulation of mRNA expression for Transforming Growth Factor-Beta1 (TGF-β1) and Vascular Endothelial Growth Factor-alpha (VEGF-α), both of which are critical biomolecules for skin renewal and angiogenesis during the healing process. Clinical studies on human burn victims indicate that beeswax mixtures can significantly reduce epithelialization initiation time and hospital duration while lowering pain scores [71].

#### 4.3.3. Anti-Inflammatory and Antipruritic Effects

The application of beeswax contributes to the reduction in inflammation and associated symptoms in dermatological disorders. It has shown efficacy in treating inflammatory conditions such as atopic dermatitis and psoriasis, leading to significant improvements in clinical signs and potentially reducing the need for corticosteroids [72]. Additionally, beeswax possesses antipruritic (anti-itch) properties; in studies regarding post-burn care, beeswax-based creams reduced itch frequency and prolonged the time before itch recurrence more effectively than standard aqueous creams [73].

#### 4.3.4. Metabolic and Gastroprotective Effects

Beeswax serves as the source of D-002, a bioactive mixture of high molecular weight alcohols (policosanols), which exhibits distinct pharmacological activities. These compounds have demonstrated anti-ulcer activity and protective effects on the gastrointestinal mucosa against experimentally induced injury [72]. Furthermore, beeswax-derived policosanols are recognized for their metabolic biofunctionality, specifically their ability to lower low-density lipoprotein (LDL) cholesterol and inhibit cholesterol synthesis, offering potential protective effects against cardiovascular diseases such as atherosclerosis [74].

## 5. Safety, Toxicity, and Regulatory Aspects

Beeswax is widely considered a non-toxic and safe substance, supported by toxicological evaluations indicating a low acute toxicity profile with an oral LD_50_ greater than 5 g/kg in rats and a lack of mutagenic or carcinogenic potential. Because of its high melting point and resistance to hydrolysis by digestive enzymes, beeswax is largely indigestible and remains unabsorbed in the mammalian gastrointestinal tract, acting primarily as an inert material [75]. In topical applications, it is generally non-irritating and possesses low comedogenic potential, making it a staple in cosmetics and skincare [4]. Although adverse dermatological reactions such as contact cheilitis have been reported, studies suggest that beeswax allergy is rare and often attributable to contaminants within the wax, such as propolis or resins, rather than the beeswax itself [76].

Regulatory bodies worldwide have validated these safety credentials, with the U.S. FDA designating yellow and white beeswax as “Generally Recognized as Safe” (GRAS) for use in food and packaging, and the European Union authorizing it as food additive E901 [75]. Despite this regulatory approval, the purity of commercial beeswax is a significant concern due to the widespread practice of adulteration [10]. Furthermore, the lipophilic nature of beeswax allows it to accumulate residues from lipophilic acaricides and environmental pesticides, creating a risk of contamination transfer when the wax is recycled for use in pharmaceutical or food industries.

Another important safety concern in beeswax is pesticide contamination, as studies have detected multiple residues (e.g., acaricides and insecticides) at levels from trace amounts (below 10 µg/kg) to 218.57 µg/kg, highlighting their persistence, accumulation, and potential for chronic exposure [77]. Although pharmaceutical-grade beeswax undergoes purification and systemic exposure is generally low due to its limited absorption, concerns remain regarding chronic exposure and the accumulation of lipophilic contaminants. Notably, no specific pharmacopeial maximum residue limits (MRLs) are currently established for beeswax as a pharmaceutical excipient. Safety is therefore managed through raw material sourcing, purification processes, and general regulatory frameworks, highlighting an important gap in standardization.

## 6. Sustainability, Challenges, and Future Directions

Beeswax is a biodegradable and environmentally benign natural material, making it an attractive sustainable alternative to synthetic waxes in cosmetic and pharmaceutical applications. Its sustainability profile, however, is strongly dependent on beekeeping practices. In traditional or fixed-comb beekeeping systems, which are still prevalent in parts of Africa and Asia, honey harvesting involves removing the entire comb, resulting in a relatively high beeswax yield, with an approximate honey-to-wax ratio of 10:1. In contrast, modern frame-hive beekeeping systems are designed to maximize honey production by reusing combs, thereby reducing wax yields to approximately 1:75. This disparity explains why a substantial proportion of European beeswax imports originates from developing countries, where traditional practices persist and provide important socioeconomic benefits to local communities [78,79]. In parallel, sustainability is being enhanced through innovations in processing technologies, including microwave-assisted extraction using ethanol, which enables more efficient recovery of beeswax components such as policosanols with reduced extraction time, lower energy demand, and diminished solvent-related environmental burdens [12]. Ethical sourcing has also become increasingly important, with greater emphasis on transparent supply chains, animal welfare considerations, and fair compensation for beekeepers [80].

Despite these advantages, the beeswax industry faces significant challenges related to quality assurance, safety, and evolving market dynamics. Economic adulteration remains the most critical issue, with beeswax frequently mixed with lower-cost materials such as paraffin, stearin, tallow, or carnauba wax. High levels of paraffin adulteration have been reported, leading to reduced melting points and compromised mechanical integrity of wax combs, which can cause structural collapse and adversely affect brood development. Detection of such adulteration is challenging because conventional physicochemical parameters, including acid and ester values, often lack sufficient sensitivity to identify low-level contamination [14]. GC/GC–MS and FTIR are widely used for the detection of beeswax adulteration, particularly with paraffin. GC-based methods are highly sensitive and can detect paraffin at levels as low as approximately 1–3% (*w*/*w*), based on characteristic changes in hydrocarbon profiles, such as the increased presence of even-numbered n-alkanes. In contrast, FTIR (including ATR-FTIR) provides rapid and non-destructive screening but exhibits higher detection limits, typically around 3–5% (*w*/*w*), depending on spectral resolution and the application of chemometric analysis. Below these levels, reliable detection may be challenging, indicating the need for complementary or more advanced analytical techniques [81]. Advanced analytical techniques such as nuclear magnetic resonance (NMR) and isotope ratio mass spectrometry (IRMS) offer enhanced specificity for beeswax authentication. NMR enables detailed molecular fingerprinting of wax constituents, while IRMS can distinguish petroleum-derived paraffin from natural beeswax based on stable carbon isotope ratios. However, these methods are less commonly applied in routine pharmaceutical quality control due to higher cost, limited accessibility, and the need for specialized expertise. They are therefore better suited as complementary tools for high-resolution authentication and origin tracing [82].

In addition, beeswax readily accumulates lipophilic chemical residues, including acaricides used for Varroa mite control, agricultural pesticides, and wood preservatives [83]. As beeswax is repeatedly recycled into comb foundations, these contaminants can concentrate over time and potentially migrate into honey, posing risks to both bee health and food safety. From a market perspective, the growing demand for vegan and plant-based cosmetic products represents a further challenge, encouraging formulators to substitute beeswax with alternative waxes and compelling producers to highlight ethical and sustainable production practices to remain competitive.

Future directions for beeswax research and application are increasingly focused on advanced analytical control [82,84], biomedical innovation [62,85], and regulatory harmonization [86]. To address fraud and ensure authenticity, the industry is progressively adopting sophisticated analytical techniques, such as gas chromatography–mass spectrometry [87] and Fourier-transform infrared spectroscopy [82] with attenuated total reflectance, which provide high sensitivity for detecting adulterants and characterizing hydrocarbon profiles. Isotope ratio mass spectrometry is also emerging as a valuable tool for determining geographic origin based on stable isotope signatures. Beyond quality control, beeswax is gaining prominence in advanced pharmaceutical and biomedical formulations, particularly in solid lipid nanoparticles [88], where it serves as a lipid matrix to improve the solubility, stability, and controlled release of lipophilic drugs, as well as to enhance skin barrier function in topical systems [34]. Concurrently, renewed interest in the bioactive components of beeswax has driven research into therapeutic applications, including policosanol-rich fractions for cardiovascular health [89] and beeswax-based formulations for wound healing, burn treatment, and inflammatory skin disorders [4]. To support these developments, there is a pressing need for comprehensive international regulatory frameworks that define quality standards for beeswax, especially for comb foundations, which are often inadequately regulated despite their direct relevance to apiculture, food safety, and public health.

## 7. Conclusions

Beeswax has successfully transitioned from a staple of ancient medicine to a sophisticated, multifunctional material in modern pharmaceutical sciences. Its evolution from a simple stiffening agent in traditional balms to a critical component in advanced DDS highlights a remarkable versatility rooted in its unique physicochemical properties, such as thermoplasticity and biocompatibility. Beyond its traditional role in stabilizing emulsions and modifying the texture of topicals, beeswax is now being utilized as a solid lipid matrix in NLCs, microspheres, and 3D-printed dosage forms. These modern applications leverage the wax to modulate drug release profiles, protect sensitive active ingredients, and improve the bioavailability of lipophilic compounds, effectively transforming beeswax from a passive vehicle into an active tool for precision medicine.

Furthermore, the inherent biofunctionality of beeswax including documented antimicrobial, anti-inflammatory, and wound-healing properties adds a significant therapeutic dimension to its pharmaceutical utility. It actively contributes to tissue regeneration and skin barrier enhancement, making it an ideal candidate for sustainable, eco-friendly dermatological formulations. However, as the industry moves toward more advanced applications, addressing challenges such as economic adulteration and the accumulation of lipophilic contaminants remains paramount. By integrating rigorous analytical techniques and establishing comprehensive international quality standards, beeswax can maintain its status as a reliable, high-performance natural excipient that bridges the gap between traditional formulation wisdom and cutting-edge biomedical innovation.

## Figures and Tables

**Table 1:** Major Chemical Constituents of Beeswax [19].

Component	Approximate Percentage (% *w*/*w*)	Key Characteristics/Significance
Linear wax monoesters and hydroxymonoesters	35–45%	Composed of long chains (C40–C48). These esters are crucial for the structural integrity and physical properties of the wax.
Complex wax esters	15–27%	Includes esters containing 15-hydroxypalmitic acid or diols.
Aliphatic hydrocarbons	12.0–17.8%	Primarily saturated hydrocarbons (n-alkanes) with an odd number of carbon atoms (predominantly C27–C33), which contribute to the wax’s hydrophobicity.
Free fatty acids	12–14%	Mainly saturated fatty acids with chain lengths of C24–C32. These acids contribute to the characteristic acid value of the wax.
Other minor substances	Varies	Includes free fatty alcohols and over 50 different aroma compounds that give beeswax its pleasant, honey-like scent.

**Table 2:** Pharmacopoeial specifications for beeswax (JP, PhEur, USP).

Test	JP XVIII	PhEur 11.0	USP47-NF42
Characters	+	+	—
Melting range	60–67 °C	61–66 °C	62–65 °C
Relative density	—	≈0.960	—
Acid value	5–9 or 17–22	17–24 (White)/17–22 (Yellow)	17–24
Ester value	—	70–80	72–79
Ester value:acid value ratio	—	3.3:4.3	—
Saponification value	80–100	87–104 (White)/87–102 (Yellow)	—
Ceresin, paraffins, and certain other waxes	—	+	—
Purity	+	—	—
Glycerols and other polyols (as glycerol)	—	+ (White)/≤0.5% (Yellow)	—
Saponification cloud test	—	—	+
Fats or fatty acids, Japan wax, rosin, and soap	—	—	+

**Table 3:** Methods for the preparation of beeswax microspheres and microcapsules.

Method	Principle/Process	Common Uses
Melt dispersion (Figure 2a)	Beeswax is melted, mixed with the core material, then rapidly cooled to solidify into microspheres.	Simple encapsulation, food, pharma [49]
Emulsion congealing (Figure 2b)	Active ingredient emulsified in molten beeswax and then cooled, causing solid microspheres to form.	Food, pharmaceuticals [39,50]
Solvent evaporation (Figure 2c)	Beeswax dissolved in organic solvent, emulsified in water, solvent evaporated to solidify wax.	Controlled drug delivery [51]
Coacervation-phase Separation (Figure 2d)	Beeswax forms a coating by phase separation, encapsulating the core on cooling or pH change.	Drugs, volatile actives [52]
Double emulsion (Figure 2e)	A water-in-oil-in-water (W/O/W) emulsion is formed by dispersing an aqueous drug phase into molten beeswax, followed by re-emulsification in an external aqueous phase and rapid cooling to solidify nanoparticles	Encapsulation of hydrophilic and hydrophobic drugs in SLN; improved drug protection and reduced cytotoxicity [53]

**Table 4:** Composition, particle characteristics, and therapeutic applications of beeswax-based nanostructured lipid carriers (NLCs).

NLC Composition	Active Ingredients	Particle Size (nm)	Applications/Therapeutic Use
Beeswax, Ethyl Oleate, Tween 80, Poloxamer 188	Naringenin and ferulic acid	271 ± 3 nm (PDI = 0.459; Zeta potential = −37.1 mV)	Topical treatment for diabetic foot ulcers; enhanced antioxidant activity; improved skin permeation (3.5×); non-irritant and biocompatible formulation [57]
Beeswax, Medium Chain Triglycerides (MCTs), Surfactant (Tween 80)	Vitamin E (α-Tocopherol)	180 ± 20 nm (PDI = 0.11 ± 0.02)	Controlled release cosmetic antioxidant; 70% release within 6 h; photoprotective and anti-aging agent for dermal delivery; viscous nanosuspension [58]
Beeswax and carvacrol	Carvacrol (5-isopropyl-2-methylphenol)	Nanoscale potential confirmed by lattice spacing increase (d = 7.25–8.50 nm)	Antimicrobial, antioxidant, and anti-inflammatory applications; demonstrated miscibility and crystallinity reduction with beeswax; suitable for green NLC systems [59]
Beeswax, Argan Oil, Ceramide NG, Cholesterol, Polysorbate 80, Glyceryl Monostearate	Ceramides and cholesterol	215.5 ± 0.9 nm (PDI = 0.25 ± 0.02; ζ = −42.7 ± 0.9 mV)	Barrier repair and hydration: an NLC hydrogel (hyaluronic acid 1%, xanthan gum 0.5–2%) providing 2.8-fold higher skin hydration, acceptable biocompatibility, and shear-thinning behavior for dermal use [60]
Beeswax, Pomegranate Seed Oil (PSO), Tween 80/Lecithin	Punicic acid (from PSO)	~203 nm (range: 71–366 nm) PDI: 0.14–0.36; ζ: −18 to −27 mV)	Food-grade antioxidant carrier system; delivers polyunsaturated fatty acids with enhanced oxidative stability; long-term stable for ≥40 days at 4 °C [43]
Beeswax, MCT, Emulsifiers (PHOSPHOLIPON^®^ 90G, Tween^®^ 80)	Curcumin	164.4 ± 10.6 nm (PDI = 0.108 ± 0.015; ζ = −11.2 ± 1.3 mV)	Improved curcumin bioaccessibility and permeability versus SLN and NE, with good digestive stability and only mild post-digestion cytotoxicity [61].

**Table 5:** Representative applications of beeswax in advanced drug delivery systems.

Type of Drug Delivery Systems	Model Drugs	Benefit of Beeswax in the System
Colon-specific delivery systems	Mesalamine	Functions as a natural coating and matrix-forming material to achieve delayed and site-specific release in the colon. Its hydrophobic structure protects the drug from upper GI degradation while promoting targeted delivery [54].
Transdermal drug delivery (Microneedle patch)	Thymol blue and Rutin	Serves as a mold material for fabricating microneedles. Beeswax’s low melting point (~62 °C), non-toxicity, and mechanical stability enable efficient, reusable mold formation without chemical residue [67]
Skin barrier enhancement	-	Incorporation of beeswax-based nanoparticles into topical formulations enhances skin hydration and barrier integrity. Clinical studies report reduced transepidermal water loss (TEWL) and improved stratum corneum hydration compared to unloaded formulations [4,34]
Oral films	Betamethasone	When blended with hydrophilic polymers (e.g., PVA, HPMC), beeswax forms flexible films capable of modulating drug dissolution. These films improve patient compliance, particularly in pediatric and geriatric populations [44,68].
3D print tablets	Fenofibrate	Acts as a biocompatible lipid carrier in hot-melt 3D inkjet printing, providing a solvent-free, thermally stable platform. Beeswax’s thermoplasticity ensures precise drug deposition and controlled release without the need for toxic solvents [40]

## Data Availability

Data sharing is not applicable due to no new data were created.

## Abbreviations

The following abbreviations are used in this manuscript:

AN	Acid Number
BHT	Butylated Hydroxytoluene
DDSs	Drug Delivery Systems
EN	Ester value
PhEur	European Pharmacopoeia
FDA	Food and Drug Administration
FTIR	Fourier-Transform Infrared Spectroscopy
GC	Gas Chromatography
GC-MS	Gas Chromatography–Mass Spectrometry
GI	Gastrointestinal
GRAS	Generally Recognized as Safe
HPMC	Hydroxypropyl Methylcellulose
IHC	International Honey Commission
JP	Japanese Pharmacopoeia
LD_50_	Median Lethal Dose
LDL	Low-Density Lipoprotein
MCT	Medium Chain Triglycerides
mRNA	Messenger Ribonucleic Acid
NLCs	Nanostructured Lipid Carriers
O/W	Oil-in-Water
PDI	Polydispersity Index
PVA	Polyvinyl Alcohol
SLNs	Solid Lipid Nanoparticles
SN	Saponification Number
TEWL	Transepidermal Water Loss
TGF-β1	Transforming Growth Factor Beta 1
USP	United States Pharmacopeia
VEGF-α	Vascular Endothelial Growth Factor Alpha
W/O	Water-in-Oil
